# Chlorhexidine-impregnated sponge versus chlorhexidine gel dressing for short-term intravascular catheters: which one is better?

**DOI:** 10.1186/s13054-020-03174-0

**Published:** 2020-07-23

**Authors:** Niccolò Buetti, Stéphane Ruckly, Carole Schwebel, Olivier Mimoz, Bertrand Souweine, Jean-Christophe Lucet, Jean-François Timsit

**Affiliations:** 1 University of Paris, INSERM IAME, U1137, Team DesCID, Paris, France; 2 Médecine Intensive Réanimation, C.H.U de Grenoble-Alpes, Grenoble, France; 3 INSERM UMR S 1039, Radiopharmaceutiques Biocliniques, Faculté de Médecine de Grenoble, Domaine de la Merci, 38700 La Tronche, France; 4 Services des Urgences Adultes and SAMU 86, Centre Hospitalier Universitaire de Poitiers, Poitiers, France; 5 Université de Poitiers, Poitiers, France; 6 INSERM U1070, Poitiers, France; 7 Medical ICU, Gabriel-Montpied University Hospital, Clermont-Ferrand, France; 8 AP-HP, Infection Control Unit, Bichat-Claude Bernard University Hospital, 46 rue Henri Huchard, 75877 Paris Cedex, France; 9 Medical and Infectious Diseases Intensive Care Unit, AP-HP, Bichat-Claude Bernard University Hospital, 46 rue Henri Huchard, 75877 Paris Cedex, France

**Keywords:** Chlorhexidine dressing, Chlorhexidine-gluconate impregnated dressing, Catheter-related infection, Catheter-related bloodstream infections, Chlorhexidine-impregnated sponges

## Abstract

**Background:**

Chlorhexidine-gluconate (CHG) impregnated dressings may prevent catheter-related bloodstream infections (CRBSI). Chlorhexidine-impregnated sponge dressings (sponge-dress) and gel dressings (gel-dress) have never been directly compared. We used the data collected for two randomized-controlled trials to perform a comparison between sponge-dress and gel-dress.

**Methods:**

Adult critically ill patients who required short-term central venous or arterial catheter insertion were recruited. Our main analysis included only patients with CHG-impregnated dressings. The effect of gel-dress (versus sponge-dress) on major catheter-related infections (MCRI) and CRBSI was estimated using multivariate marginal Cox models. The comparative risks of dressing disruption and contact dermatitis were evaluated using logistic mix models for clustered data. An explanatory analysis compared gel-dress with standard dressings using either CHG skin disinfection or povidone iodine skin disinfection.

**Results:**

A total of 3483 patients and 7941 catheters were observed in 16 intensive care units. Sponge-dress and gel-dress were utilized for 1953 and 2108 catheters, respectively. After adjustment for confounders, gel-dress showed similar risk for MCRI compared to sponge-dress (HR 0.80, 95% CI 0.28–2.31, *p* = 0.68) and CRBSI (HR 1.13, 95% CI 0.34–3.70, *p* = 0.85), less dressing disruptions (OR 0.72, 95% CI 0.60–0.86, *p* < 0.001), and more contact dermatitis (OR 3.60, 95% CI 2.51–5.15, *p* < 0.01). However, gel-dress increased the risk of contact dermatitis only if CHG was used for skin antisepsis (OR 1.94, 95% CI 1.38–2.71, *p* < 0.01).

**Conclusions:**

We described a similar infection risk for gel-dress and sponge-dress. Gel-dress showed fewer dressing disruptions. Concomitant use of CHG for skin disinfection and CHG-impregnated dressing may significantly increase contact dermatitis.

**Trials registration:**

These studies were registered within ClinicalTrials.gov (numbers NCT01189682 and NCT00417235).

## Background

Short-term central venous catheters (CVCs) are instrumental in the care of critically ill patients for the intravenous administration of fluid resuscitation, safe intravenous administration of medications, and support in the monitoring of hemodynamic parameters. While the CVC utilization rate was on average 70 CVC days per 100 patient days, 44% of the ICU-bloodstream infections (BSIs) were related to a catheter [1]. Catheter-related infections were associated with high morbidity and mortality [2]. Several prevention measures are well known in the literature [3–7]. Among them, chlorhexidine-impregnated dressings were beneficial in various studies to prevent catheter-related bloodstream infections [8, 9] and were currently recommended by the Centers for Disease Control and Prevention (CDC) [10]. Two types of chlorhexidine-impregnated dressing are currently used: chlorhexidine-impregnated sponges (sponge-dress) [11] and chlorhexidine gel dressing (gel-dress) [12]. Both sponge-dress and gel-dress reduced the rate of intravascular catheter-related infections [11, 12]. Sponge-dress does not allow the continuous inspection of the insertion site and is difficult to apply; however, it showed very low rate of contact dermatitis. To our knowledge, a direct comparison between sponge-dress and gel-dress has never been performed. An extensive prospective high-quality data collection was performed for two large RCTs [11, 12], and these data were used for this post hoc study to compare the rates of intravascular catheter infections, dressing disruptions, and contact dermatitis between sponge-dress and gel-dress.

## Material and methods

### Study design

We used the databases from two large RCTs (DRESSING1 and DRESSING2 studies) that both investigated chlorhexidine-impregnated dressings versus standard dressings [11, 12]. The similarities among these RCTs with regard to definitions and inclusion criteria allowed us to merge the two databases. The DRESSING1 study investigated the impact of sponge-dress and frequency of dressing changes for preventing catheter-related infections or catheter colonization [11]. The DRESSING2 study assessed the effect of gel-dress and highly adhesive dressing for preventing catheter-related infections and catheter colonization [12]. Both sponge-dress and gel-dress decreased the rate of intravascular catheter infection. The studies interventions were neither blinded to the investigators nor to the ICU staff, but they were blinded to the adjudication committee and to the microbiologists who processed the samples of blood and catheter cultures. Both studies were approved by the national ethic committees; further ethical consent was not required according to the French law for research. Both RCTs complied with CONSORT guidelines and the current analysis complied with the STROBE guidelines for observational studies [13, 14].

### Study patients

Adult patients (≥ 18 years) who required a CVC or a peripheral arterial catheter (AC) insertion were recruited from 2006 to 2011 in 16 intensive care units (ICU) in France. Among all participating ICUs, three participated to both studies. The characteristics of patients were similar across studies. Patients underwent follow-up until 48 h after ICU discharge or death.

### Study catheters and dressings

This post hoc analysis evaluated data from patients with short-term CVCs and ACs included in both studies. All catheters were managed similarly and complied with the French recommendations for catheter insertion and care, which are similar to CDC guidelines [15]: (1) maximal sterile barrier precautions were used (large sterile drape; surgical hand antisepsis; and mask, cap, sterile gloves, and gown); (2) the site of insertion was selected at the discretion of the physician caring for the patient; (3) povidone-iodine solution (PVI) or alcoholic ≤ 0.5% chlorhexidine gluconate (CHG) was used for skin antisepsis at catheter insertion and during dressing changes at the discretion of the physician or according to ICU policies; (4) dressings were used regardless of the insertion sites and were changed 24 h after catheter insertion and then every 3 or 7 days OR according to standard practice in each ICU. Leaking, soiled, or wet dressings were changed immediately. None of the study catheters was antibiotic- or antiseptic-impregnated. Of note, in the DRESSING1 study, CHG was rarely used for skin disinfection. Decisions to remove catheters were made independently by the physicians caring for each patient.

### Definitions and outcomes

According to American and French recommendations, we used the following definitions [16, 17]: catheter colonization was defined as a quantitative catheter tip culture yielding ≥ 1000 colony-forming units (cfu)/mL [18]. A catheter-related bloodstream infection (CRBSI) was a combination of (i) one or more positive peripheral blood cultures sampled 48 h before or after catheter removal; (ii) the isolation of the same phenotypic microorganism from the colonized catheter or a blood culture differential time-to positivity of 2 h or more [19]; and (iii) no apparent source of bloodstream infection other than the catheter. Catheter-related clinical sepsis without bloodstream infection was a combination of catheter colonization, body temperature (≥ 38·5 °C or ≤ 36·5 °C), pus at the insertion site, or resolution of clinical sepsis after catheter removal, and the absence of any other infectious focus. MCRI was defined as either a CRBSI or a catheter-related clinical sepsis without bloodstream infection. If a patient had a positive blood culture for coagulase-negative staphylococci (CoNS), the same pulsotype from the strains recovered from the catheter tip and blood culture was required for a diagnosis of a CRBSI. Alternatively, two separate peripheral blood cultures had to grow the same microorganism that colonized the catheter tip.

Dressing disruption was defined by a leakage or soiling and led to an immediate dressing change.

The condition of the skin was described on a standardized form by the nurse in charge of the patient at each dressing change and at catheter removal, using the International Contact Dermatitis Research Group system (ICDRC; 0, normal skin; 1, mild redness only; 2, red and slightly thickened skin; 3, intense redness and swelling with coalesced large blisters or spreading reaction). We created a binary variable called contact dermatitis (ICDRC ≥ 1 versus without contact dermatitis with ICDRC = 0).

### Statistical analysis

Characteristics of patients and catheters were described as count (percent) or median (interquartile range) for qualitative and quantitative variables, respectively.

The statistical plan had three objectives: (1) to identify the risk differences in MCRI and CRBSI between sponge-dress and gel-dress using catheters as statistical unit, (2) to evaluate the risk of dressing disruption between sponge-dress and gel-dress, and (3) to determine the risk of contact dermatitis between sponge-dress and gel-dress, using “dressings” as statistical unit for these longitudinal data. To achieve our objectives, we included in our main analysis only CHG-impregnated dressings, and we excluded Tegaderm HP® and standard dressings.

For the first objective, we used a marginal Cox model for clustered data to take into account a possible clustering effect of multiple catheters per patient. This model takes into account the possible intra-cluster dependence using a robust sandwich covariate estimate and the censored nature of the data. Analyses were stratified by catheter type (CVC versus AC), and we censored the data at 28 days since catheter insertion. Hazard risk for MCRI and CRBSI was evaluated by univariate and multivariate analyses. The variable “dressing” (sponge-dress versus gel-dress) was forced in our multivariate models, and other well-known risk factors for MCRI were used as adjustment factors (i.e., sex, mechanical ventilation at admission, experience of the operator, insertion site). As CHG skin disinfection was predominantly used in the DRESSING2 study, we performed a sensitivity analysis for the catheters inserted using either PVI or CHG. The proportionality of hazard risks for gel-dress (versus sponge-dress) was tested using Martingale residuals.

For the second and third objective, we used mixed logistic models for clustered data (PROC GLIMMIX of SAS) with the response variable “dressing disruption” or “contact dermatitis,” respectively, and we adjusted for the time between catheter insertion and dressing change, and the ICU. These models take into account a possible clustering effect of multiple dressings per catheter. Our group previously analyzed risk factors for dressing disruption: therefore, we performed a sensitivity analysis adjusting for well-known dressing disruption risk factors (i.e., gender, chronic renal failure, coma as main reason for ICU admission and subclavian site). For contact dermatitis, we performed a sensitivity analysis using as outcome an ICDRC ≥ 2. Moreover, we performed an explanatory analysis for the DRESSING2 study considering only catheters inserted with CHG skin disinfection or catheters inserted with only PVI skin disinfection. For all models, we performed a sensitivity analysis with solely the three ICUs which participated in both studies.

Tests were two-tailed, with *p* < 0.05 being considered significant. All analyses were performed using SAS (version 9.4; SAS Institute, Cary, NC) and R (version 3.5.3). Informed consent was obtained from all individual participants included in the study and whose decision-making capacity was intact.

## Results

### Patients, catheters, and dressings

**Fig. 1:**
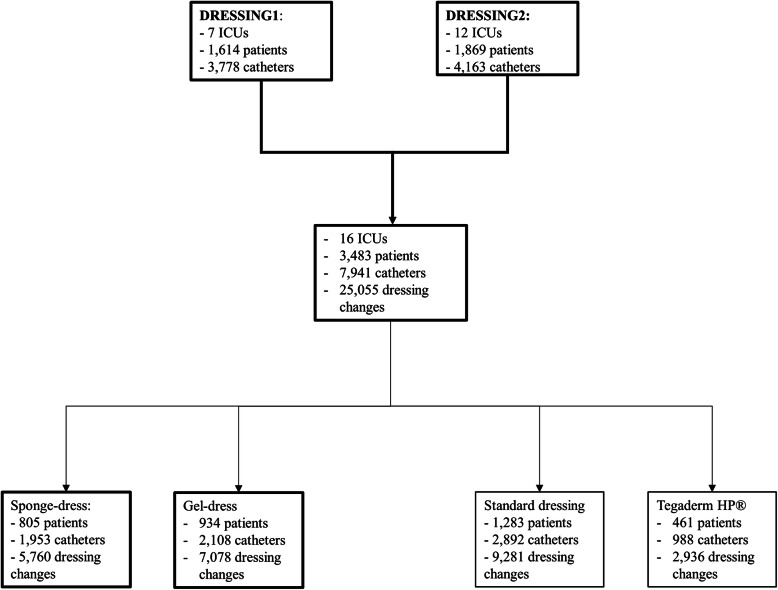
Flow-chart. ICU: Intensive care unit. Sponge-dress: Chlorhexidine-impregnated sponges. Gel-dress: Chlorhexidine-impregnated dressing. Tegaderm HP®: highly adhesive dressing

Between 2006 and 2011, a total of 3483 patients, 7941 catheters, and 25,055 dressing changes were observed (Fig. 1).

Sponge-dress and gel-dress were utilized for 1953 and 2108 catheters, respectively, whereas Tegaderm HP® and standard dressing were used in 988 and 2862 catheters, respectively.

Characteristics of patients and catheters with CHG-impregnated dressings were described in Tables 1 and 2. The patients were similar between both groups, except for the reasons for ICU admission which were different between sponge-dress and gel-dress.

**Table 1:** Characteristics of patients with CHG-impregnated dressings

	Gel-dress (*n* = 934)	Sponge-dress (*n* = 805)
Male, *n* (%)	635 (68)	526 (65.3)
Age, median (IQR)	63.5 [53; 74]	62 [50; 74]
No comorbidity, *n* (%)	636 (68.1)	558 (69.3)
Cancer, *n* (%)	62 (6.6)	31 (3.9)
Chronic renal failure, *n* (%)	38 (4.1)	34 (4.2)
Chronic cardiac failure, *n* (%)	74 (7.9)	34 (4.2)
Chronic respiratory failure, *n* (%)	39 (4.2)	52 (6.5)
Diabetes mellitus, *n* (%)	52 (5.6)	62 (7.7)
Immunosuppression, *n* (%)	82 (8.8)	82 (10.2)
Hemopathy or hematological malignancy, *n* (%)	31 (3.3)	23 (2.9)
Reason for ICU admission, *n* (%)		
Shock	317 (33.9)	334 (41.5)
Coma	87 (9.3)	108 (13.4)
Trauma	55 (5.9)	93 (11.6)
Respiratory failure	265 (28.4)	176 (21.9)
Other	210 (22.5)	94 (11.7)
SAPS II, median (IQR)	52 [39; 68]	52 [40; 65]
Mechanical ventilation at admission, *n* (%)	680 (72.8)	676 (84)
Vasopressor at admission, *n* (%)	553 (59.2)	572 (71.1)
In-ICU mortality, *n* (%)	292 (31.3)	269 (33.4)

Legends. *IQR* interquartile range, *ICU* intensive care unit, *Sponge-dress* chlorhexidine-impregnated sponges, *Gel-dress* chlorhexidine-impregnated dressing, *SAPS* Simplified Acute Physiology Score

**Table 2:** Characteristics of catheters with CHG-impregnated dressings

	Gel-dress (*n* = 2108)	Sponge-dress (*n* = 1593)
Catheter days, median (IQR)	5 [3; 10]	5 [3; 9]
CVC	980 (46.5)	1056 (54.1)
Experience of the operator < 50 procedures	764 (36.2)	1396 (71.5)
Insertion site for CVC		
Jugular	275 (28.1)	312 (29.5)
Subclavian	332 (33.9)	412 (39)
Femoral	373 (38.1)	332 (31.4)
Insertion site for AC		
Femoral	393 (34.8)	353 (39.4)
Radial	735 (65.2)	544 (60.6)
Skin antisepsis with CHG	1533 (72.7)	20 (1)
Mechanical ventilation at insertion	1616 (76.7)	1688 (86.4)
Vasopressor at insertion	959 (45.5)	1222 (62.6)
Antibiotics at insertion	1183 (56.1)	1324 (67.8)
Suspicion of infection	278 (13.2)	342 (17.5)
Catheter colonization	71 (3.4)	92 (4.7)
MCRI	10 (0.5)	9 (0.5)
CRBSI	7 (0.3)	5 (0.3)

Legends. *IQR* interquartile range, *Sponge-dress* chlorhexidine-impregnated sponges, *Gel-dress* chlorhexidine-impregnated dressing, *CVC* central venous catheter, *AC* arterial catheter, *CHG* chlorhexidine-gluconate, *MCRI* major catheter-related infections, *CRBSI* catheter-related bloodstream infections

Skin antisepsis was performed with CHG (*n* = 20) for only few catheters with sponge-dress, whereas more experienced operators inserted catheters in the gel-dress group. Catheter colonization was slightly increased in the sponge-dress group.

### MCRI and CRBSI risk

**Fig. 2:**
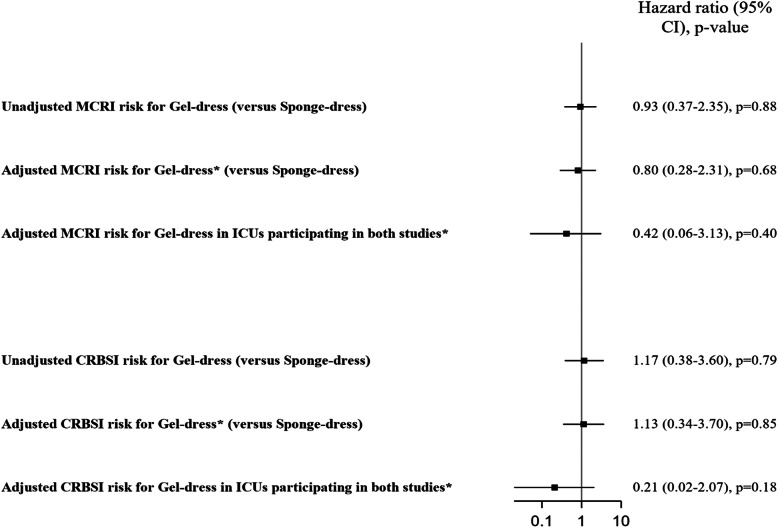
Unadjusted and adjusted MCRI and CRBSI risk using marginal Cox models. Legend. MCRI: major catheter-related infection. CRBSI: catheter-related bloodstream infection. Sponge-dress: chlorhexidine-impregnated sponges. Gel-dress: chlorhexidine-impregnated dressing. *Adjustment variables were sex, mechanical ventilation at admission, experience of the operator, and insertion site. For the adjusted analyses for gel-dress in ICUs participating in both studies, we performed an additional analysis stratifying for ICU and we observed similar results for MCRI (HR 0.30, 95% CI 0.06–1.48, *p* = 0.14) and CRBSI (HR 0.16, 95% CI 0.02–1.70, *p* = 0.13)

The proportionality of hazard risks for gel-dress (versus sponge-dress) was respected for MCRI (*p* = 0.98) and CRBSI (*p* = 0.45). In the univariate Cox model, the risk for MCRI (HR 0.93, CI 95% 0.37–2.35, *p* = 0.88) and CRBSI (HR 1.17, CI 95% 0.38–3.60, *p* = 0.79) was similar in the gel-dress group compared to those of the sponge-dress group (Fig. 2, Supplementary material Tables S1-S2).

After adjustment for confounders, gel-dress showed similar risk compared to sponge-dress for MCRI (HR 0.80, 95% CI 0.28–2.31, *p* = 0.68) and CRBSI (HR 1.13, 95% CI 0.34–3.70, *p* = 0.85). A sensitivity analysis for the 3 ICU which participated in both studies showed similar results. Among catheters inserted with PVI (*n* = 2508), the risk for MCRI (HR 1.80, 95% CI 0.55–5.92, *p* = 0.33) and CRBSI (HR 2.25, 95% CI 0.44–11.45, *p* = 0.33) was statistically not different between gel-dress and sponge-dress (data not shown).

### Dressing disruptions among CHG-impregnated dressings

**Fig. 3:**
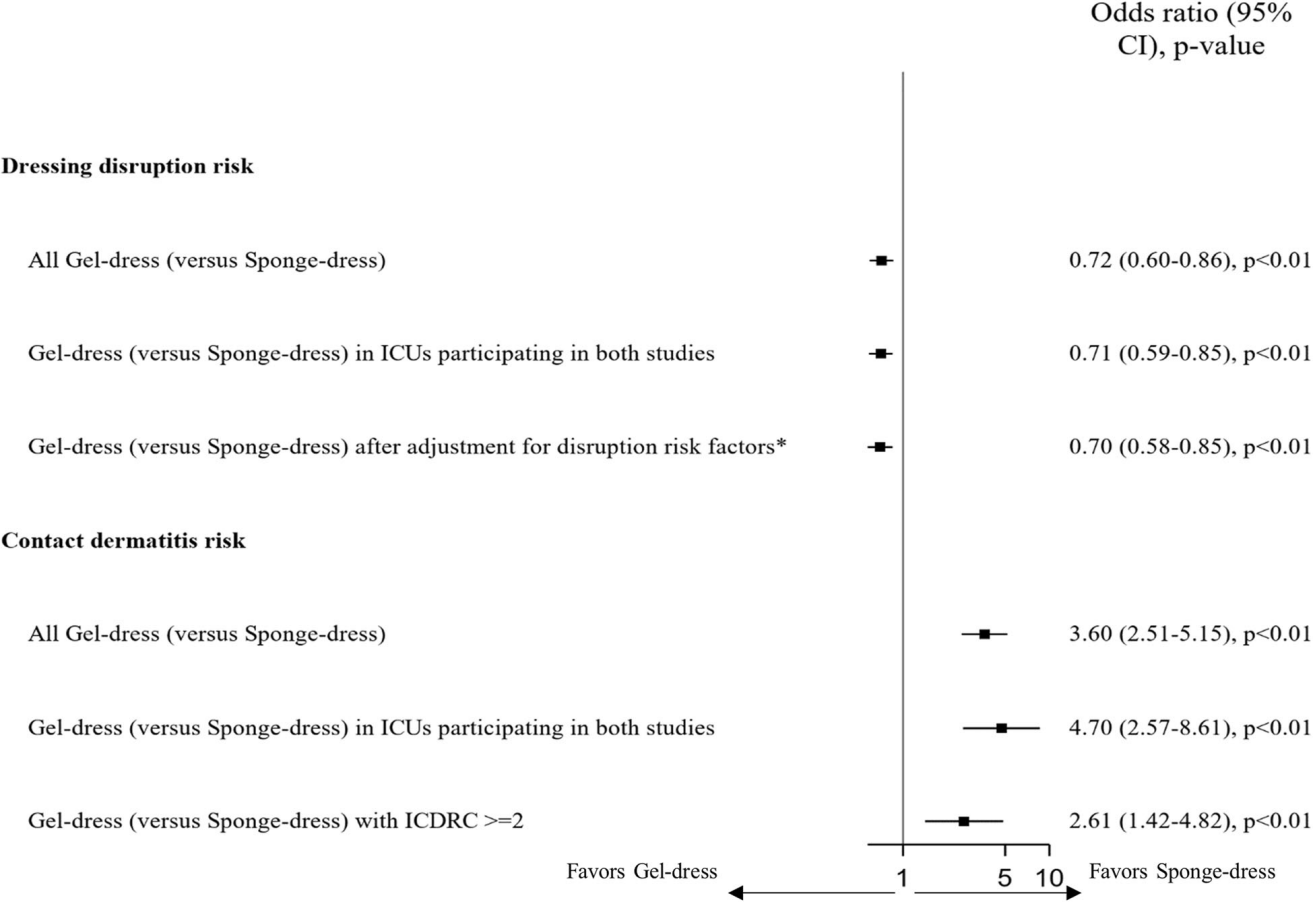
Dressing disruption and contact dermatitis risk. Legend. Sponge-dress: Chlorhexidine-impregnated sponges. Gel-dress: Chlorhexidine-impregnated dressing. CI: Confidence interval. ICU: Intensive care unit. ICDRC: International Contact Dermatitis Research Group system. *Adjustment variables were sex, chronic renal failure, coma at admission, SOFA score, and subclavian site

We observed 5760 dressing changes and 3761 dressing disruption (65.3%) in sponge-dress, whereas 7078 changes and 4946 disruptions (69.9%) were observed in gel-dress. Dressing disruption were infrequently observed in gel-dress compared to sponge-dress (OR 0.72, 95% CI 0.60–0.86, *p* < 0.001, Fig. 3).

Considering only the three ICUs included in both studies (OR for gel-dress 0.71, CI 95% 0.59–0.85, *p* < 0.001) or after adjustment with disruption risk factors, we observed similar results (OR 0.70, 95% CI 0.58–0.85, *p* < 0.01, Fig. 3).

### Contact dermatitis

We observed 100 (1.7%) and 609 (8.6%) contact dermatitis in sponge-dress and gel-dress, respectively. Gel-dress had an increased risk for contact dermatitis compared to sponge-dress (OR 3.60, 95% CI 2.51–5.15, *p* < 0.01, Fig. 3). Considering only the three ICUs included in both studies, we observed similar results (OR for gel-dress 4.70, CI 95% 2.57–8.61, *p* < 0.01). Considering contact dermatitis with ICDRC ≥ 2, we observed 23 (0.4%) and 194 (2.7%) contact dermatitis in sponge-dress and gel-dress, respectively; the mixed model showed similar results (OR for gel-dress 2.61, CI 95% 1.42–4.82, *p* < 0.01).

An explanatory analysis using only data of the DRESSING2 study and catheters inserted with PVI skin antisepsis showed that gel-dress did not significantly increase the risk for contact dermatitis compared to standard dressings (OR 1.40, 95% CI 0.83–2.36, *p* = 0.21, data not shown). In contrast, if CHG was used for skin antisepsis, gel-dress increased the risk of contact dermatitis compared to standard dressing (OR 1.94, 95% CI 1.38–2.71, *p* < 0.01, data not shown).

## Discussion

Using high-quality data from two RCTs, this post hoc analysis showed that the daily hazard rate of intravascular catheter infections was similar between gel-dress and sponge-dress. We observed fewer dressing disruptions among gel-dress compared to sponge-dress, whereas sponge-dress was associated with fewer contact dermatitis.

A recent meta-analysis showed that chlorhexidine-impregnated dressings reduced catheter-related bloodstream infections [9]. To our knowledge, a direct comparison between gel-dress and sponge-dress has never been performed. A recent “real-world data study” comparing gel-dress and sponge-dress data used in different time periods confirmed that the addition of CHG dressings to existing catheter bundles provided a significant decrease in the rate of CRBSI [20]. This study showed that a non-significant lower rate of infections occurred with gel-dress compared with sponge-dress, while our data clearly demonstrated a similar rate of infections between both types of CHG-impregnated dressings. Therefore, we confirmed the data obtained in vitro by Karpanen et al. that showed similar antimicrobial activity of gel-dress compared to sponge-dress [21].

Dressing disruptions were less frequently observed among gel-dress. This may be explained by the difficulty to appropriately use sponge-dress. Moreover, sponge-dress may fail to contact the skin around the catheter insertion site if the fixation sutures for catheter were very near the entry point [12]. Even if not confirmed by our MCRI and CRBSI analyses, dressing disruptions may be an important risk factor for catheter-related infections and should therefore be prevented [22].

Contact dermatitis was more frequently observed when gel-dress was used compared to sponge-dress. This result should be interpreted with caution. An explanatory analysis of the DRESSING2 (i.e., including only gel-dress and standard dressings) study showed that contact dermatitis in gel-dress was significantly increased (compared to standard dressing) only if CHG was used for the skin antisepsis. Using PVI skin disinfection, gel-dress did not significantly increase the risk of dermal reaction (OR of 1.4, *p* = 0.21). Therefore, we hypothesize that contact dermatitis was triggered by the cumulative exposition to CHG (i.e., CHG used for skin disinfection *and* CHG-impregnated dressings).

In light of these considerations, gel-dress appeared to have a slight benefit in terms of dressing disruptions compared to sponge-dress. Moreover, gel-dress permitted a continuous inspection of the insertion site that may help clinicians in managing intravascular catheters [23]. A particular attention to contact dermatitis should be paid if CHG is used for skin disinfection and concomitantly CHG-impregnated dressing was applied.

Our study has several limitations. First, we performed an observational study, and unmeasured factors may cause residual confounding. However, we presented high-quality data that were prospectively collected during both RCTs. Second, not all ICUs were included in both studies, and the experience of the operator and the skin antisepsis used was different between the two studies. However, we performed a sensitivity analysis for the three ICUs included in both DRESSING1 and DRESSING2 studies, and it showed the same results as the primary analysis. Moreover, we adjusted our Cox models for the experience of the operator and skin antisepsis mode. Third, sponge-dress and gel-dress were used in two different periods, thus liming the comparison between the two groups. However, with the exception of CHG skin antisepsis, the prevention policies did not change between the two study periods. Of note, prevention strategies did not change from 2011, except the current use of alcoholic 2% CHG for skin antisepsis. Finally, for the allergy analysis, we observed low rates of outcomes in several subgroups, and therefore, the mix models were simplified. Moreover, as the interpretation of the outcome ICDRC ≥ 1 is clinically debatable, we performed a sensitivity analysis using ICDRC ≥ 2, which showed comparable results.

## Conclusions

Using the largest dataset ever collected from large multicentered RCTs conducted with consistent catheter care, we illustrated that the infection risk was similar for gel-dress and sponge-dress. Gel-dress showed fewer dressing disruptions. Concomitant use of CHG for skin disinfection and CHG-impregnated dressing may significantly increase contact dermatitis.

## Supplementary information

**Additional file 1: Table S1.** Univariate and multivariate marginal Cox models for MCRI. **Table S2.** Univariate and multivariate marginal Cox models for CRBSI.

## Data Availability

The datasets used and/or analyzed during the current study are available from the corresponding author on reasonable request.

## Abbreviations

AC: Arterial catheter
BSI: Bloodstream infection
CHG: Chlorhexidine-gluconate
CI: Confidence interval
CVC: Central venous catheter
CRBSI: Catheter-related bloodstream infection
CoNS: Coagulase-negative staphylococci
Gel-dress: Chlorhexidine-impregnated gel dressing
HR: Hazard ratio
ICU: Intensive care unit
MCRI: Major catheter-related infection
RCT: Randomized-controlled trial
SAPS: Simplified Acute Physiology Score
Sponge-dress: Chlorhexidine-impregnated sponge dressing
