# Supplementary material for: Chlorhexidine-impregnated sponge versus chlorhexidine gel dressing for short-term intravascular catheters: which one is better?
Source: Crit Care. 2020 Jul 23;24:458. doi: 10.1186/s13054-020-03174-0 (PMC7376730; doi:10.1186/s13054-020-03174-0)
Supplement: Supplementary file 1 — Additional file 1: Table S1. Univariate and multivariate marginal Cox models for MCRI. Table S2. Univariate and multivariate marginal Cox models for CRBSI. [file 13054_2020_3174_MOESM1_ESM.docx]

**Supplementary material**

**Table S1: Univariate and multivariate marginal Cox models for MCRI.**

|  |  | No MCRI (n=4042) | MCRI (n=19) | HR | 95% CI | p-value | HR | 95% CI | p-value |
| --- | --- | --- | --- | --- | --- | --- | --- | --- | --- |
|  |  | **Univariate marginal Cox model** | | |  |  | **Multivariate marginal Cox model** | | |
| Gel-dress (*versus* Sponge-dress) |  | **2,098 (51.9)** | **10 (52.6)** | **0.933** | **[0.37-2.35]** | **0.8826** | **0.80** | **[0.28-2.31]** | **0.68** |
| Male sex |  | 2,734 (67.6) | 7 (36.8) | 0.273 | [0.11-0.69] | 0.0062 | 0.33 | [0.13-0.85] | 0.02 |
| Age, median (IQR) | | 62 [50 ; 73] | 63 [54 ; 69] | 0.999 | [0.98-1.02] | 0.9314 |  |  |  |
| No comorbidity | | 2,766 (68.4) | 13 (68.4) | 1.286 | [0.45-3.71] | 0.6420 |  |  |  |
| Chronic cardiac failure | | 236 (5.8) | 2 (10.5) | 1.829 | [0.24-13.85] | 0.5588 |  |  |  |
| Chronic respiratory failure | | 227 (5.6) | 1 (5.3) | 0.577 | [0.09-3.51] | 0.5499 |  |  |  |
| Immunosuppression | | 407 (10.1) | 2 (10.5) | 0.841 | [0.2-3.47] | 0.8107 |  |  |  |
| SAPS II, median (IQR) | | 53 [41 ; 66] | 47 [31 ; 64] | 0.993 | [0.96-1.03] | 0.6907 |  |  |  |
| Mechanical ventilation at admission | | 3,251 (80.4) | 12 (63.2) | 0.384 | [0.16-0.93] | 0.0342 | 0.45 | [0.19-1.07] | 0.07 |
| Vasopressor at admission | | 2,801 (69.3) | 11 (57.9) | 0.690 | [0.27-1.79] | 0.4451 |  |  |  |
| CVC |  | 2,024 (50.1) | 12 (63.2) | 1.182 | [0.5-2.81] | 0.7047 |  |  |  |
| Experience of the operator > 50 procedures |  | 1,892 (46.8) | 9 (47.4) | 1.248 | [0.5-3.13] | 0.6372 | 0.78 | [0.34-1.82] | 0.5684 |
| Insertion site (reference radial) | Jugular | 582 (14.4) | 5 (26.3) | 3.829 | [0.74-19.71] |  | 4.214 | [0.53-33.63] | 0.1747 |
|  | Subclavian | 740 (18.3) | 4 (21.1) | 1.920 | [0.36-10.34] |  | 2.240 | [0.28-17.95] | 0.4474 |
|  | Femoral | 1,443 (35.7) | 8 (42.1) | 4.824 | [0.98-23.83] |  | 4.809 | [0.86-26.81] | 0.0733 |
|  | Radial | 1,277 (31.6) | 2 (10.5) |  |  | 0.1713 |  |  |  |
| Skin antisepsis with CHG ≤0.5% | | 1,548 (38.3) | 5 (26.3) | 0.499 | [0.18-1.37] | 0.1787 |  |  |  |
| Mechanical ventilation at insertion | | 3,288 (81.3) | 16 (84.2) | 0.888 | [0.26-2.99] | 0.8476 |  |  |  |
| Vasopressor at insertion | | 2,175 (53.8) | 6 (31.6) | 0.472 | [0.18-1.25] | 0.1319 |  |  |  |
| Antibiotics at insertion | | 2,496 (61.8) | 11 (57.9) | 0.657 | [0.26-1.66] | 0.3753 |  |  |  |

Legends. IQR: Interquartile range. HR: Hazard ratio. CI: Confidence interval. HR: hazard ratio. CIS: Chlorhexidine-impregnated sponges. CID: Chlorhexidine-impregnated dressing. CVC: Central venous catheter. SAPS II score: Simplified Acute Physiology Score II. CHG: chlorhexidine-gluconate. MCRI: Major catheter-related infection. The analysis was stratified by catheter type (CVC *versus* arterial catheter).

**Table S2: Univariate and multivariate marginal Cox models for CRBSI.**

|  |  | No CRBSI (n=4049) | CRBSI (n=12) | HR | 95% CI | p-value | HR | 95% CI | p-value |
| --- | --- | --- | --- | --- | --- | --- | --- | --- | --- |
|  |  | **Univariate marginal Cox model** | | |  |  | **Multivariate marginal Cox model** | | |
| Gel-dress (*versus* Sponge-dress) |  | **2,101 (51.9)** | **7 (58.3)** | **1.168** | **[0.38-3.6]** | **0.7873** | **1.125** | **[0.34-3.70]** | **0.8459** |
| Male sex |  | 2,736 (67.6) | 5 (41.7) | 0.332 | [0.11-1.01] | 0.0531 | 0.452 | [0.16-1.32] | 0.1452 |
| Age, median (IQR) | | 62 [50 ; 73] | 66 [59.5 ; 69] | 1.014 | [0.99-1.04] | 0.3294 |  |  |  |
| No comorbidity | | 2,770 (68.4) | 9 (75) | 1.787 | [0.5-6.45] | 0.3750 |  |  |  |
| Chronic cardiac failure | | 238 (5.9) | 0 (0) |  |  |  |  |  |  |
| Chronic respiratory failure | | 227 (5.6) | 1 (8.3) | 0.956 | [0.15-6.04] | 0.9616 |  |  |  |
| Immunosuppression | | 408 (10.1) | 1 (8.3) | 0.644 | [0.09-4.84] | 0.6689 |  |  |  |
| SAPS II, median (IQR) | | 53 [41 ; 66] | 53.5 [32.5 ; 75.5] | 1.001 | [0.96-1.04] | 0.9642 |  |  |  |
| Mechanical ventilation at admission | | 3,257 (80.4) | 6 (50) | 0.229 | [0.08-0.67] | 0.0070 | 0.252 | [0.09-0.73] | 0.0114 |
| Vasopressor at admission | | 2,806 (69.3) | 6 (50) | 0.514 | [0.17-1.56] | 0.2405 |  |  |  |
| CVC |  | 2,028 (50.1) | 8 (66.7) | 1.357 | [0.42-4.43] | 0.6132 |  |  |  |
| Experience of the operator > 50 procedures |  | 1,895 (46.8) | 6 (50) | 1.385 | [0.46-4.2] | 0.5647 | 0.809 | [0.31-2.12] | 0.6666 |
| Insertion site (reference radial) | Jugular | 583 (14.4) | 4 (33.3) | 6.010 | [0.68-53.18] |  | 13.750 | [0.61- 310] | 0.0994 |
|  | Subclavian | 741 (18.3) | 3 (25) | 2.843 | [0.3-26.89] |  | 7.186 | [0.31- 167] | 0.2194 |
|  | Femoral | 1,447 (35.7) | 4 (33.3) | 4.781 | [0.52-43.89] |  | 5.741 | [0.55-59.49] | 0.1430 |
|  | Radial | 1,278 (31.6) | 1 (8.3) | . |  | 0.3818 |  |  |  |
| Skin antisepsis with CHG ≤0.5% | | 1,549 (38.3) | 4 (33.3) | 0.692 | [0.21-2.23] | 0.5375 |  |  |  |
| Mechanical ventilation at insertion | | 3,295 (81.4) | 9 (75) | 0.501 | [0.14-1.79] | 0.2878 |  |  |  |
| Vasopressor at insertion | | 2,177 (53.8) | 4 (33.3) | 0.509 | [0.15-1.69] | 0.2710 |  |  |  |
| Antibiotics at insertion | | 2,499 (61.7) | 8 (66.7) | 0.937 | [0.29-3.02] | 0.9132 |  |  |  |

Legends. IQR: Interquartile range. HR: Hazard ratio. CI: Confidence interval. HR: hazard ratio. CIS: Chlorhexidine-impregnated sponges. CID: Chlorhexidine-impregnated dressing. CVC: Central venous catheter. SAPS II score: Simplified Acute Physiology Score II. CHG: chlorhexidine-gluconate. CRBSI: Catheter-related bloodstream infection. The analysis was stratified by catheter type (CVC *versus* arterial catheter).
